# Bacterial Vaginosis Is Associated with Loss of Gamma Delta T Cells in the Female Reproductive Tract in Women in the Miami Women Interagency HIV Study (WIHS): A Cross Sectional Study

**DOI:** 10.1371/journal.pone.0153045

**Published:** 2016-04-14

**Authors:** Maria L. Alcaide, Natasa Strbo, Laura Romero, Deborah L. Jones, Violeta J. Rodriguez, Kristopher Arheart, Octavio Martinez, Hector Bolivar, Eckhard R. Podack, Margaret A. Fischl

**Affiliations:** 1 Division of Infectious Diseases, Department of Medicine, University of Miami Miller School of Medicine, Miami, Florida, 33136, United States of America; 2 Department of Microbiology and Immunology, University of Miami Miller School of Medicine, Miami, Florida, 33136, United States of America; 3 Department of Psychiatry and Behavioral Sciences, University of Miami Miller School of Medicine, Miami, Florida, 33136, United States of America; 4 Department of Epidemiology and Public Health, University of Miami Miller School of Medicine, Miami, Florida, 33136, United States of America; 5 Department of Pathology, University of Miami Miller School of Medicine, Miami, Florida, 33136, United States of America, Hector Bolivar, Division of Infectious Diseases, Department of Medicine, University of Miami Miller School of Medicine, Miami, Florida, 33136, United States of America; Fred Hutchinson Cancer Center, UNITED STATES

## Abstract

Bacterial vaginosis (BV) is the most common female reproductive tract infection and is associated with an increased risk of acquiring and transmitting HIV by a mechanism that is not well understood. Gamma delta (GD) T cells are essential components of the adaptive and innate immune system, are present in the female reproductive tract, and play an important role in epithelial barrier protection. GD1 cells predominate in the mucosal tissue and are important in maintaining mucosal integrity. GD2 cells predominate in peripheral blood and play a role in humoral immunity and in the immune response to pathogens. HIV infection is associated with changes in GD T cells frequencies in the periphery and in the female reproductive tract. The objective of this study is to evaluate if changes in vaginal flora occurring with BV are associated with changes in endocervical GD T cell responses, which could account for increased susceptibility to HIV. Seventeen HIV-infected (HIV+) and 17 HIV-uninfected (HIV-) pre-menopausal women underwent collection of vaginal swabs and endocervical cytobrushes. Vaginal flora was assessed using the Nugent score. GD T cells were assessed in cytobrush samples by flow cytometry. Median Nugent score was 5.0 and 41% of women had abnormal vaginal flora. In HIV uninfected women there was a negative correlation between Nugent score and cervical GD1 T cells (b for interaction = - 0.176, p<0.01); cervical GD1 T cells were higher in women with normal vaginal flora than in those with abnormal flora (45.00% vs 9.95%, p = 0.005); and cervical GD2 T cells were higher in women with abnormal flora than in those with normal flora (1.70% vs 0.35%, p = 0.023). GD T cells in the genital tract are protective (GD1) and are targets for HIV entry (GD2). The decrease in cervical GD1 and increase in GD2 T cells among women with abnormal vaginal flora predisposes women with BV to HIV acquisition. We propose to use GD T cell as markers of female genital tract vulnerability to HIV.

## Introduction

Bacterial Vaginosis (BV) is the most common female reproductive tract (FRT) infection and occurs in up to 40% of women at risk of or infected with the Human Immunodeficiency Virus (HIV) [1–5]. BV is characterized by an increase in the vaginal pH, reduction in vaginal hydrogen-peroxide producing lactobacilli and an increase in gram negative anaerobic bacteria. BV impacts reproductive health as it is associated with numerous adverse gynecological outcomes such as preterm delivery, complications of gynecological surgeries and pelvic inflammatory disease [1, 6, 7]. Additionally, BV increases the risk of sexually transmitted infections (STI), HIV acquisition and HIV transmission to male sex partners and newborns [8–13].

The risk factors for developing BV and the mechanisms underlying the association between BV, STI and HIV remain largely unknown. Epidemiological and clinical studies have identified associations between BV and other factors, including African American ethnicity, intravaginal douching, new sexual partners, multiple sex partners, and unprotected vaginal intercourse [14, 15]. It has also been suggested that BV may be caused by a sexually transmitted pathogen (*Gardnerella vaginalis*) [16, 17]. Mechanisms by which risk factors are linked with the occurrence of BV and by which BV increases FRT vulnerability to HIV and STI have not been been completely elucidated.

Gamma delta (GD) T cells are immune cells associated with responsiveness to viral, bacterial and protozoal antigens. GD T cells are present in the peripheral blood and represent 1–10% of circulating T lymphocytes. GD cells express either the V delta 1 (GD1) or the V delta 2 (GD2) receptor. GD1 cells predominate in the mucosal tissue where they maintain epithelial tissue integrity. GD2 cells are the major GD T cell population in peripheral blood and play a role in humoral immunity and in the immune response to systemic infections. The effect of HIV infection on peripheral GD T cells has been studied previously and an increased level of GD1 T cells and decreased level of GD2 T cells suggest their important antiviral role [18, 19]. Our team has been the first to document the presence of GD1 and GD2 T cells in the endocervix of women with HIV or at risk for HIV infection. We have also shown that the majority of endocervical GD T cells are GD1 T cells, characterized by the lack of expression of both CD4 and CD8 (CD4-CD8-), whereas endocervical GD2 T cells express CD4 and the chemokine receptor CCR5 [20].

Due to the unique innate characteristics of GD T cells, which exert a direct antimicrobial and cytotoxic activity (GD T cells do not require priming for effector activity and secrete and array of different antimicrobial and growth factors) [21–23], we hypothesized that the changes in the vaginal flora occurring with BV would be associated with changes in endocervical GD T cell responses, which could clarify the pathway by which BV increases the risk of both acquiring and transmitting HIV infection.

## Materials and Methods

### Ethics statement

Approval from the Institutional Review Board (University of Miami Miller School of Medicine) was obtained prior to recruitment and any assessment or study related procedures. Participants were provided with information about the study and assured of confidentiality of study records. All participants provided voluntary written informed consent at the time of enrollment.

### Study procedures

Study activities took place at AIDS Clinical Research Unit and the Behavioral Research Unit at the University of Miami, in collaboration with the Miami Women and HIV Interagency Study (WIHS) and the Miami Center for AIDS Research (CFAR). Dates of study enrollment were from April 2014 to November 2014. Participants were women aged 18 to 45 years of age and sexually active in the 3 months prior to enrollment. Participants were excluded if they were pregnant, on contraceptive medications or with an intrauterine device. Participants were administered a web- based assessment of demographic, sexual risk factors characteristics and medical history by the study coordinator. Participants underwent a 10 milliliter blood draw and vaginal examination with collection of genital samples. This is a pilot study and no data on GD T cells in the cervical mucosa exist in the literature. Therefore, the sample was a convenience sample and sample size was not calculated.

Women without documentation of HIV status underwent HIV testing. HIV testing was performed by using the rapid test OraQuick ADVANCE^®^ Rapid HIV-1/2 Antibody Test. If positive, a confirmatory HIV western blot was performed. Women with history of HIV infection presented documentation of HIV infection (HIV western blood results, medical records, or any laboratory results with detectable HIV viral load) and had a rapid test performed to confirm HIV infection.

### Demographics and sexual risk factors

Demographic and sexual risk factors collected included: age, race and ethnicity, marital status, educational level, employment, yearly income, alcohol use in the prior month, number of partners, use of male condoms and history of exchanging sex for money.

### Medical History

Medical history collected included: history of STI (gonorrhea, syphilis, chlamydia, PID, herpes, trichomoniasis), history of BV, history of candida vaginitis and date from last menstrual period.

Among women with HIV infection, use of antiretrovirals was self-reported. Plasma CD4 T cells/millimeter and HIV RNA viral load were measured as part of the study.

### Genital tract specimen collection

A vaginal swab and cervical brush were collected in the following order: first a vaginal swab was collected and vaginal secretions were placed on a microscopy slide, then the cervical os was visualized and cleaned of mucous. A Cytobrush^®^ Plus GT cell collector was then inserted into the endocervix until the bottom-most bristles were exposed, rotated 360, removed and inserted in a 15 mL tube containing 5 mL of IMDM and placed on ice. Genital findings on examination, such as presence of lesions, characteristics of vaginal discharge, vaginal pH, cervical lesions, ectopy or friability, were noted.

### Sexually transmitted infections

Chlamydia and gonorrhea infections were diagnosed using nucleic acid amplification testing (Becton Dickinson ProbeTec^®^ Chlamydia and Gonorrhea Displacement Assay) on endocervical swabs.

### Bacterial Vaginosis

BV was diagnosed using Nugent criteria. Abnormal vaginal flora was defined as Nugent score of 4 or above. A diagnosis of BV was made when the Nugent score was 7 or above. Slides of vaginal secretions were analyzed by Gram stain at the University of Miami microbiology laboratories. Laboratory staff received training prior to scoring slides and the same trained laboratory technician scored all the slides. In case of unclear scoring, the slide was reviewed by a second technician and the chief of the laboratory until an agreement was achieved.

### Endocervical cytobrush processing

The cytobrush sample was processed within one hour of collection and the extracted cells were combined for staining and flow cytometric analysis. Tubes with 5 ml IMDM (Iscove Modified Dulbecco’s Media) and cytobrush were vortexed for 1 min then centrifuged with the cytobrush in the tube. The cytobrush was then taken out from the tube, placed on a strainer and washed with additional 20 ml of (IMDM). After straining, the cell suspension was pipetted up and down several times to break up any remaining mucus. The pellet from the first 5 ml of IMDM and the additional cytobrush wash were combined and centrifuged for 10 min at 250g. The supernatant was carefully decanted and cells resuspend in 1 ml of IMDM and counting was performed using a Beckman Coulter automated cell counter (The Vi-CELL Series Cell Viability Analyzer) with the trypan blue dye exclusion method (total viable cell concentration were reported).

### Staining and flow cytometry acquisition

Cells resuspended in FACS buffer (PBS containing 1% bovine serum albumin [Sigma]) were transferred to 5 mL round bottom, snap cap tubes (BD^®^) and stained with the LIVE/DEAD Fixable Aqua Dead Cell Stain kit (Life Technologies^®^, Grand Island, NY, USA). Cells were washed with FACS wash (PBS containing 1% bovine serum albumin [Sigma]) and stained with the antibody panel for phenotyping (CD45, CD3, CD4, CD8, CD195, TCR GD2, TCR GD1, TCR αβ, all from Biolegend, BD Pharmingen and Thermo Scientific). Cells were washed again with FACS buffer, resuspended in 300 mL 1% paraformaldehyde. Antibody panel optimization and titrations were performed in cells isolated from cytobrushes. Compensation controls were prepared simultaneously with sample processing, using UltraComp eBeads, (eBioscience^®^) for antibodies and ArC Amine Reactive Compensation Beads (Life Technologies^®^) for the viability stain. Samples were acquired on Fortessa flow cytometer (BD^®^), equipped with 405 nm, 488 nm, and 635 nm lasers. Forward and side scatter voltages were normalized using Trucount beads and fluorescence parameter PMTs were normalized by use of Rainbow Calibration Particles, Peak 7 (Spherotech^®^, Lake Forest, IL, USA).

### Data analysis

Data was collected using Qualtrics software version 61093 (Qualtrics^®^ Provo, UT) and laboratory data was entered into an excel spreadsheet. Data from both sources was converted and merged into a single file using IBM SPSS Statistics version 22.0 (SPSS^®^), Armonk, NY running on a Windows 7 operating system. Descriptive analyses were conducted to describe demographic, medical, sexual risk factors, Nugent scores, BV and endocervical GD T cell frequencies (percentage of endocervical GD T cells within CD3+ T cells). Sociodemographic and medical history comparisons by HIV status were conducted using t tests or its nonparametric alternative (Mann-Whitney U test) in the case of continuous variables. For categorical variables, a chi-square or Fisher’s exact test was used. The dependent variables were the frequency of GD1 T cells, and GD2 T cells in the cervical brushes. Normality assumption was assessed by examining histograms and indices of skewness and kurtosis for the dependent variables (endocervical GD1 and GD2 T cells). Given the moderate positive skew of the data, a square root transformation was applied to GD1 T cells, rendering a normal distribution. Similarly, given the severe positive skew of the data, GD2 T cells were logarithmically transformed, resulting in a normal distribution. Independent variables included demographics, sexual risk factors, medical history, Nugent score, BV and HIV status. To test whether Nugent score predicts frequency of GD1 and GD2 T cells, and whether this varies as a function of HIV status, two multiple linear regressions were conducted with transformed GD1 T cells as dependent variable and Nugent score and HIV status and their interaction as the independent variables. A similar model was fit using transformed GD2 T cells as the dependent variable. To assess for differences in GD1 and GD2 T cells across different cutoffs of Nugent scores (i.e., abnormal vaginal flora and BV), HIV status, and their interaction, analyses of variance (ANOVAs) were conducted. A 2 (HIV+, HIV-) x 2 (normal vaginal flora: Nugent less than 4, abnormal vaginal flora: Nugent 4 or greater) ANOVA was conducted to test for differences in GD1 T cells by HIV status, presence of abnormal vaginal flora, and the interaction of HIV status and vaginal flora. Similar tests were used to test for differences in GD2 T cells, changing only the dependent variable from GD1 T cells to GD2 T cells in the previously described analyses. Significant interaction effects were followed up with post hoc pairwise comparisons with a Bonferroni correction. A p-value of less than 0.05 was considered to be significant.

## Results

### Sociodemographic characteristics and medical history

Details of sociodemographic characteristics and medical history by HIV status are illustrated in Table 1.

**Table 1:** Sociodemographic characteristics and medical history (N = 34).

Characteristic	All	HIV- (n = 17)	HIV + (n = 17)	X^2^/t/Z, p
	n (%)	n (%)	n (%)	
	Mean (SD)	Mean (SD)	Mean (SD)	
Age	34.1 (6.2)	36.1 (6.4)	32.2 (5.5)	1.89, 0.068
Race				
Black/African American	25 (73.5%)	11 (44.0%)	14 (56.0%)	
White	7 (20.6%)	5 (71.4%)	2 (28.6%)	
Mixed or other	2 (5.9%)	1 (50.0%)	1 (50.0%)	1.81, 0.688^1^
Ethnicity				
Hispanic	9 (26.5%)	7 (77.8%)	2 (22.2%)	
Non-Hispanic	18 (52.9%)	8 (44.4%)	10 (55.6%)	
Haitian	4 (11.8%)	0 (0.0%)	4 (100.0%)	
Other	3 (8.8%)	2 (66.7%)	1 (33.3%)	7.00, 0.055^1^
Marital Status				
Never married	16 (47.1%)	7 (43.8%)	9 (56.3%)	
Stable partnership	10 (29.4%)	7 (70.0%)	3 (30.0%)	
Unstable partnership	5 (14.7%)	2 (40.0%)	3 (60.0%)	2,02, 0.442^1^
Educational level				
High school or less	12 (35.3%)	8 (66.7%)	4 (33.3%)	
More than high school	21 (61.8%)	9 (42.9%)	12 (57.1%)	1.73, 0.282
Employed				
Yes	10 (32.3%)	5 (50.0%)	5 (50.0%)	
No	21 (67.7%)	11 (52.4%)	10 (47.6%)	0.015 0.999^1^
Yearly Income (USD)				
≤ 1200	21 (61.8%)	**7 (33.3%)**	**14 (66.7%)**	
> 1200	10 (29.4%)	**9 (90.0%)**	**1 (10.0%)**	**8.71, 0.006**^1^
Alcohol use last month				
Yes	15 (46.9%)	10 (66.7%)	5 (33.3%)	
No	14 (43.8%)	6 (37.5%)	10 (62.5%)	2.64, 0.156
Number of partners prior 5 yrs	12.0 (21.2)	19.1(27.9)	4.9 (6.22)	1.74, 0.085^2^
Condom use past month				
Always	12 (37.5%)	5 (41.7%)	7 (58.3%)	
Sometimes	7 (21.9%)	4 (57.1%)	3 (42.9%)	
Never	13 (40.6%)	8 (61.5%)	5 (38.5%)	1,10, 0.660^1^
History of sex for compensation				
Yes	14 (42.0%)	9 (64.3%)	5 (35.7%)	
No	17 (58.0%)	8 (42.1%)	11 (57.9%)	1.59, 0.296
History of gonorrhea				
Yes	4 (11.8%)	0 (0.0%)	4 (100.0%)	
No	30 (88.2%)	17 (56.7%)	13 (43.3%)	4.53, 0.103^1^
History of syphilis				
Yes	4 (11.8%)	1 (25.0%)	3 (75.0%)	
No	30 (88.2%)	16 (53.3%)	14 (46.7%)	1.33, 0.601^1^
History of chlamydia				
Yes	13 (38.2%)	5 (38.5%)	8 (61.5%)	
No	21 (61.8%)	12 (57.1%)	8 (42.9%)	1.12, 0.481
History of PID				
Yes	4 (11.8%)	1 (25.0%)	3 (75.0%)	
No	30 (88.2%)	16 (53.3%)	14 (46.7%)	1,13, 0.601^1^
History of genital herpes				
Yes	5 (14.7%)	2 (40.0%)	3 (60.0%)	
No	29 (85.3%)	15 (51.7%)	14 (48.3%)	0.23, 0.999^1^
History of trichomoniasis				
Yes	9 (26.5%)	4 (44.4%)	5 (55.6%)	
No	29 (73.5%)	13 (52.0%)	12 (48.0%)	0.15, 0.999^1^
History of bacterial vaginosis				
Yes	10 (29.4%)	4 (40.0%)	6 (60.0%)	
No	24 (70.6%)	13 (54.2%)	11 (45.8%)	0.57, 0.708
History of candida vaginitis				
Yes	17 (50.0%)	**12 (70.6%)**	**5 (29.4%)**	
No	17 (50.0%)	**5 (29.4%)**	**12 (70.6%)**	**5.77, 0.038**

Note.

^1^Fischer’s exact test was used.

^2^Mann-Whitney U test was used to compare groups. PID = pelvic inflammatory disease.

Participants were 17 HIV-infected (HIV+) and 17 HIV-uninfected (HIV-) at risk, pre-menopausal women. Participant’s sociodemographic characteristics and risk factors did not differ by HIV status with the exception of yearly income, which was higher in women without HIV infection. Mean age was 34.1 years (*SD* = 6.2). Participants were primarily black/African American race (25, 73.5%) and non-Hispanic ethnicity (18, 52.9%). Almost half of the women had never been married. Most women had education of high school level or less (21, 61.8%), were unemployed (21, 67.7%) and had a yearly income less than 12,000 USD (21, 61.8%). Approximately half (15, 46.9%) of the women reported having used alcohol in the last month. Both HIV infected and uninfected women had risk factors for STIs and HIV infection: the number of self-reported sexual partners was high (mean of 12 in the prior 5 years), rates of condom use were low (37.5% of women reported 100% condom use in the prior month) and almost half (42.0%) had a history of exchanging sex for compensation (money, gifts or drugs).

Rates of prior STIs per lifetime (gonorrhea, syphilis, chlamydia, PID, genital herpes or trichomoniasis) were high and did not differ by HIV status. Rates of prior vaginal infections (BV and candida vaginitis) were also high and HIV- women reported having lower rates of candida vaginitis. The median time from the first day of the last menstrual cycle was 18.5 days (SD = 7.4) and was not statistically different in HIV- vs HIV+ women (day 16 of the cycle for HIV- and day 19 for HIV+ women; not shown in table).

The majority of HIV-infected women were on antiretroviral medications (12, 70.5%). The median CD4 count was 493 cells per milliliter (*SD* = 321.9). Seven participants (41.2%) had detectable plasma viremia as defined as more than 20 copies per milliliter (median viral load = 4.36 log_10_, SD = 1.6; not shown in table).

### Genital examination

Genital examination was unremarkable in the majority of participants: five participants had increased vaginal discharge of normal characteristics, one participant had an off white/greyish discharge and three had friability of the cervix. The mean pH high (5.3) and was similar in HIV- and HIV+ women (Table 2). Two HIV- participants tested positive for STI (one was positive for chlamydia and one for gonorrhea).

**Table 2:** pH, Nugent score, vaginal flora, bacterial vaginosis and frequency of GD T cells by HIV status (N = 34).

Variable	All	HIV- (n = 17)	HIV + (n = 17)	X^2^/t/Z, p
	n (%)	n (%)	n (%)	
	Mean (SD)	Mean (SD)	Mean (SD)	
pH (n = 20)	5.3 (0.85)	4.9 (0.78)	5.5 (0.82)	1.81, 0.086
Nugent score (n = 33)	4.6 (3.2)	3.8 (3.3)	5.5 (3.0)	1.59, 0.121
Bacterial Vaginosis				
Yes	10 (29.4%)	4 (40.0%)	6 (60.0%)	
No	24 (70.6%)	13 (54.2%)	11(45.8%)	0.57, 0.708
Abnormal vaginal flora				
Yes	20 (58.8%)	8 (40.0%)	12 (60.0%)	
No	14 (41.2%)	9 (64.3%)	5 (35.7%)	1.94, 0.296
GD1 T cells	25.3 (25.7)	**38.0 (28.2)**	**14.3 (18.1)**	**2.28, 0.022**^1^
GD2 T cells	1.02 (1.54)	**1.1 (0.9)**	**0.91 (2.0)**	**2.29, 0.024**^1^

Note.

^1^Mann-Whitney U test was used to compare groups. GD: gammadelta

### Nugent Criteria, bacterial vaginosis and Gamma delta T cells in endocervical cytobrushes

Nugent score, BV rates, rates of abnormal vaginal flora and frequency of GD T cells by HIV status are illustrated in Table 2.

Mean Nugent scores were: 4.6 (*SD* = 3.2), almost one third of the women had BV, and more than half had abnormal vaginal flora as defined by Nugent score of 4 or above. No differences in Nugent scores, rates of BV or rates of abnormal vaginal flora were found when comparing women by HIV serostatus.

Mean frequency of GD1 T cells in cervical cytobrushes was 25.33 (*SD* = 25.66); and mean frequency of endocervical GD2 T cells was 1.02 (*SD* = 1.54). As previously described by our group, [20] we found statistically significant decrease in the frequency of endocervical GD1 and GD2 T cells in HIV uninfected women compared to infected women (GD1 in HIV- = 38.0% vs GD1 in HIV+ = 14.3%, p = 0.022; and GD2 HIV- = 1.1% vs GD2 in HIV+ = 0.91%, p = 0.024).

### Changes in the vaginal flora are associated with depletion of endocervical GD1 T cells in HIV- women

**Fig 1:**
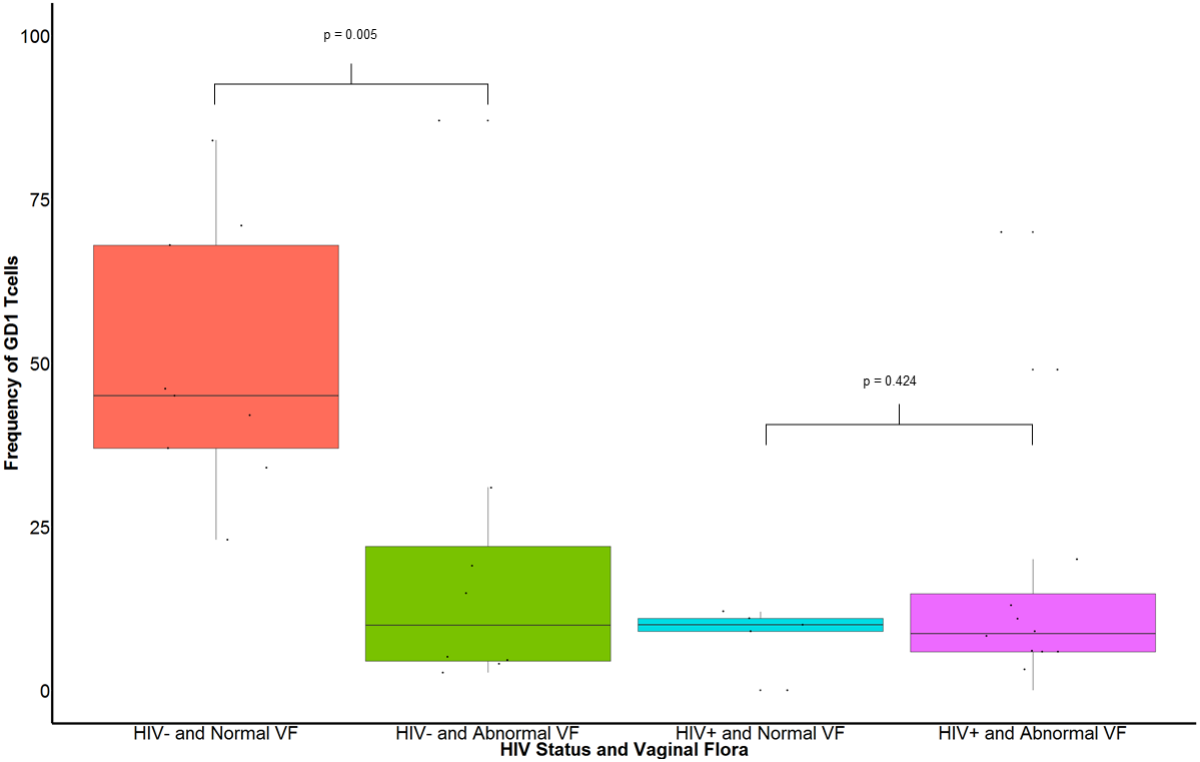
Comparison of percentages of endocervical GD1 T cells in HIV infected and uninfected women with normal and abnormal vaginal flora. VF: vaginal flora. Normal VF is defined as Nugent score less than 4. Abnormal VF is defined as Nugent score of 4 or greater than 4. In HIV- women, frequency of GD1 T cells was higher in women with normal vaginal flora than in those with abnormal vaginal flora (45.00 vs 9.95, p = 0.005). Results from a 2 (HIV+, HIV-) x 2 (normal VF, abnormal VF) ANOVA showed that there was a nonsignificant main effect of abnormal vaginal flora, *F*(1,33) = 2.12, *p* = 0.155, but a statistically significant main effect of HIV status, *F*(1,33) = 10.34, *p* = 0.003. The interaction of BV and HIV status was statistically significant, *F*(1,33) = 7.05, *p* = 0.013. Further analysis of the interaction effect of BV and HIV status using pairwise comparisons with a Bonferroni correction showed that there was a statistically significant difference in GD1 T cells among HIV- women with abnormal vaginal flora compared to those without abnormal vaginal flora (*p* = 0.005), but not among HIV+ women (*p* = 0.424).

Comparison of frequency of endocervical GD1 T cells in HIV+ and HIV- women with normal and abnormal vaginal flora is illustrated in Fig 1. In HIV- women, frequency of endocervical GD1 T cells was higher in women with normal vaginal flora than in those with abnormal vaginal flora (45.00% in HIV- with normal vaginal flora vs 9.95% in women HIV- with abnormal vaginal flora, p = 0.005) (Fig 1).

**Fig 2:**
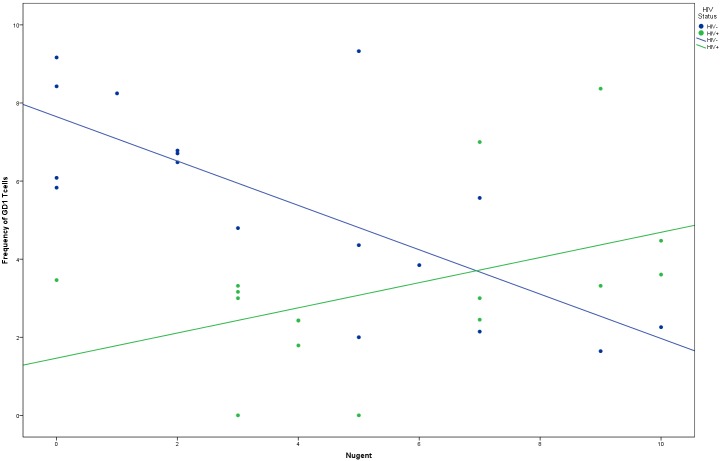
Interaction of Nugent score and HIV status predicting GD1 T cells. Results of the regression model predicting endocervical GD1 T cells by HIV status, Nugent score, and the interaction of HIV status and Nugent score showed that for HIV- participants, GD1 T cells decreased as Nugent score increased, but for HIV+ women, as Nugent score increased, GD1 T cells decreased (b for interaction = 1.197, *p* < 0.001 for HIV- women)

Results of the regression model predicting endocervical GD1 T cells by HIV status, Nugent score, and the interaction of HIV status and Nugent score showed that for HIV- women, GD1 T cells increased as Nugent score increased, but for HIV+ women, as Nugent score increased, GD1 T cells decreased (b for interaction = 1.197, *p* < 0.001 for HIV- women) (Fig 2).

### Changes in the vaginal flora are associated with increase of endocervical GD2 T cells in HIV- women

**Fig 3:**
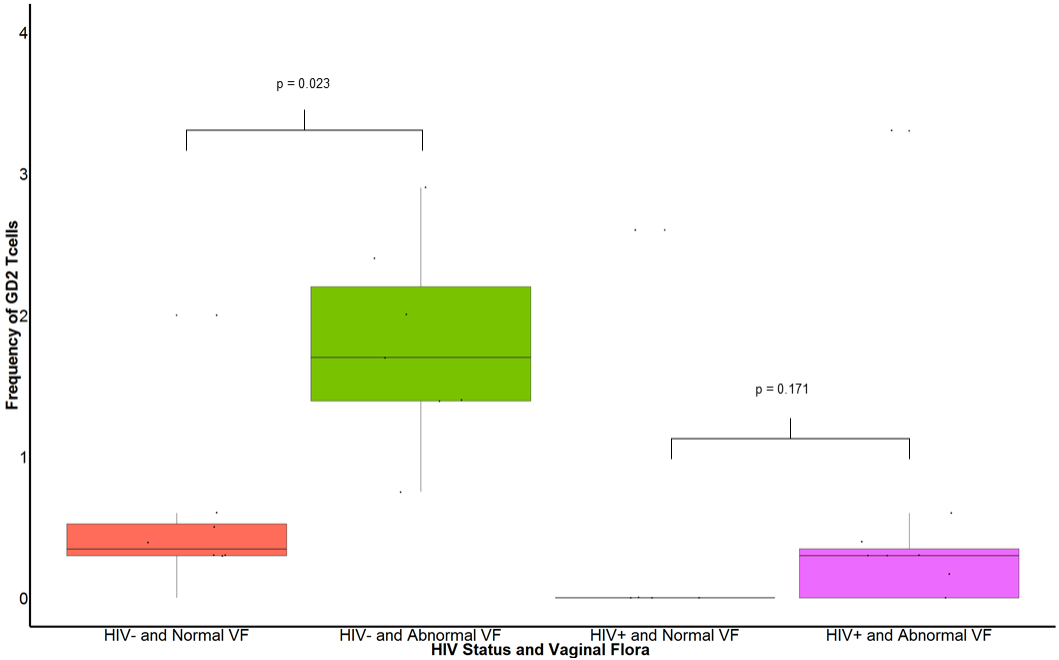
Comparison of frequencies of endocervical GD2 T cells in HIV infected and uninfected women with normal and abnormal vaginal flora. VF: vaginal flora. Normal VF is defined as Nugent score less than 4. Abnormal VF is defined as Nugent score of 4 or greater than 4. In HIV- women, frequency of GD2 T cells was higher in women with abnormal vaginal flora than in those with normal vaginal flora (1.79 vs 0.55, p = 0.023). A 2 (HIV+, HIV-) x 2 (normal VF, abnormal VF) ANOVA indicated that there was a nonsignificant main effect of vaginal flora, *F*(1,22) = .03, *p* = .876, but a nonsignificant main effect of HIV status, *F*(1,22) = .43, *p* = .520. The interaction of vaginal flora and HIV status was statistically significant, *F*(1,22) = 5.67, *p* = .028. The interaction effect was further analyzed using pairwise comparisons with a Bonferroni correction, which showed that there was a statistically significant difference in GD2 T cells among HIV- women with abnormal vaginal flora (Nugent score more than 4) compared to those with normal vaginal flora (*p* = 0.023), but not among HIV+ women (*p* = 0.171).

Comparisons of frequencies of endocervical GD2 T cells in HIV+ and HIV- women who had normal or abnormal vaginal flora are illustrated in Fig 3. In HIV- women, frequency of GD2 T cells was lower in women with normal vaginal flora than in women with abnormal vaginal flora (0.55% in HIV- women with normal flora vs 1.79% in HIV- women with abnormal flora, p = 0.023).

In addition to an increased frequency, an increase absolute numbers of endocervical GD2 T cells was found in the endocervical samples with abnormal vaginal flora (0.3 x10^3^ GD2 T cells in the endocervical samples with abnormal vaginal flora vs 0.003x 10^3^ GD2 T cells in the endocervical samples with normal vaginal flora).

Results of the regression model predicting GD2 T cells by HIV status, Nugent score, and the interaction of HIV status and Nugent score showed that the interaction of HIV status and Nugent did not significantly predict GD2 T cells (b for interaction = -0.623, *p* = 0.207) (not shown in figure).

Exploratory analyses were done to evaluate if the frequencies of GD1 or GD2 T cells were associated with CD4 counts or plasma viremia in women with HIV infection. There was no association between the frequency of GD1 or GD2 T cells and CD4 counts or plasma viremia (data not shown).

## Conclusions

In this pilot study we evaluated the relationship between changes in the vaginal flora and endocervical gamma delta (GD) T cells among women enrolled in the Miami Women Interagency HIV Study (WIHS). The WIHS is one of the largest longitudinal study of women with HIV or at risk for HIV infection in the United States. We found that rates of BV were high in both HIV infected and uninfected women, and describe for the first time a relationship between abnormal vaginal flora and endocervical GD1 and GD2 T cells in women at risk for HIV infection.

The deleterious health consequences of abnormal vaginal flora and the association of abnormal vaginal flora with increased HIV acquisition have been well documented but the mechanism has not been completely elucidated. It is likely that disruption of the mucosal cells that act as a barrier to infection in the FRT induces an inflammatory milieu accompanied by an increase in pro-inflammatory cytokines and decrease in the natural antimicrobial activity that favors the growth of BV-associated bacteria (*Gardnerella vaginalis*, *Mobiluncus sp*., *Bacteroides sp*., and others) increasing vulnerability to HIV infection. [24–27] In addition, changes in the vaginal flora occurring with BV are associated with an increase HIV target cells that express CCR5 at the mucosal level. [28].

Peripheral GD T cells play a major role in immunosurveillance and expand during the acute phase of an infection. Intraepithelial mucosal GD1 T cells represent “true first line” immune effector cells capable of providing rapid surveillance responses to microbial tissue perturbations. [22] It has been previously documented that systemic bacterial infections induce expansion of peripheral GD T cells responses. [29] However, the effect of bacterial infections at mucosal surfaces on the local GD T cell responses is unknown.

Though several earlier *in vitro* studies have shown changes in the mucosal immune response and in the epithelial cells after inoculation with genital secretions of women with BV or BV associated bacteria [30–32], the current study is the first to evaluate GD T cells in the FRT and to evaluate the relationship of changes in the vaginal flora with mucosal GD T cells. As a mechanistic hypothesis, we propose that changes in vaginal flora and increases in inflammatory cytokines occuring with BV, [33] facilitate dysregulation of GD T cells at mucosal level, similarly to what as has been observed at the systemic level.

The findings of this study have important relevance for women at risk for HIV infection as they provide a novel mechanism to explain the increase in HIV acquisition associated with BV. The primary target cells for HIV acquisition via vaginal intercourse are mucosal cervical CD4 T cell lymphocytes that express receptors and coreceptors necessary to gain intracellular access (CD4 and CCR5) [34, 35], and mucosal CCR5+ cells; and CD4+ and CCR5+ cells, are increased in the presence of BV [28]. The well described regulatory phenotype of human decidual GD T cells (secretion of IL-10 and TGFbeta) clearly supports GD T cell potential to contribute to maintenance of a non-inflammatory micro-environment. [36, 37] In addition to their regulatory role, we know that GD1 cells in the blood express higher levels of the cytotoxic pore-forming protein perforin than GD2, giving them the potential for immediate cytotoxic response upon pathogen or stress encounter at the epithelial barrier. [38].

Since GD1 T cells are the predominant GD T cell subtype in the cervix, these cells likely represent a first line of defense against HIV infection in the FRT. Although associations found do not necessarily indicate a causal relationship, we believe the decrease of GD1 T cells in women with BV suggests a decrease in first line of immune defense to HIV infection. In addition, GD2 T cells are the predominant GD cells in the periphery, and an increase in both absolute number and proportion of endocervical GD2 T cells (cells that express CD4 and CCR5) likely represents recruited peripheral GD2 cells to the mucosal surfaces under BV microbe-initiated activation and an increased risk of HIV target cells in the cervix. Recruitment of GD cells from the periphery to mucosal surfaces has previously been described by Poles in women with HIV, but we are the first to suggest this as a potential explanation of how the shift in cervical GD T cells subtypes may contribute to HIV acquisition in women with BV [39].

The primary limitation of this pilot study is its small sample size as it is difficult to determine mechanisms of pathogenesis with such small numbers. As a cross sectional study, our results evaluate correlations but do not prove causality and longitudinal data could provide more context for inferring cause and effect. Future studies should include the assessment of the vaginal microbiome, molecular assays or culture of the vaginal flora, lower genital inflammatory cytokines and antimicrobial secretions, HIV lower genital shedding and the relationship with other STIs. [40–42].

Despite the limitations inherent to a cross-sectional study, our results suggest that GD T cells are important immune cells in the FRT and changes in GD associated with abnormal vaginal flora predispose women with BV to HIV acquisition. Our outcomes support the evaluation of GD T cells in studies addressing FRT vulnerability to HIV, including those assessing the safety of vaginal microbicides and other genitally applied HIV prevention methods.

### Disclaimer

Data in this manuscript were collected by the Women’s Interagency HIV Study (WIHS). The contents of this publication are solely the responsibility of the authors and do not represent the official views of the National Institutes of Health (NIH). WIHS (Principal Investigators): UAB-MS WIHS (Michael Saag, Mirjam-Colette Kempf, and Deborah Konkle-Parker), U01-AI-103401; Atlanta WIHS (Ighovwerha Ofotokun and Gina Wingood), U01-AI-103408; Bronx WIHS (Kathryn Anastos), U01-AI-035004; Brooklyn WIHS (Howard Minkoff and Deborah Gustafson), U01-AI-031834; Chicago WIHS (Mardge Cohen and Audrey French), U01-AI-034993; Metropolitan Washington WIHS (Mary Young), U01-AI-034994; Miami WIHS (Margaret Fischl and Lisa Metsch), U01-AI-103397; UNC WIHS (Adaora Adimora), U01-AI-103390; Connie Wofsy Women’s HIV Study, Northern California (Ruth Greenblatt, Bradley Aouizerat, and Phyllis Tien), U01-AI-034989; WIHS Data Management and Analysis Center (Stephen Gange and Elizabeth Golub), U01-AI-042590; Southern California WIHS (Alexandra Levine and Marek Nowicki), U01-HD-032632 (WIHS I–WIHS IV).

## Supporting Information

S1 Table De-identified data set. (XLSX)
